# Observational Study of Clinical Practice in Patients with Pancreatic Adenocarcinoma in Greece

**DOI:** 10.1155/2020/5304516

**Published:** 2020-09-19

**Authors:** George Papaxoinis, Athanasios Athanasiadis, Joseph Sgouros, Anastastios Visvikis, Maria Drizou, Emmanouil Kontopodis, Anna Koumarianou, Suzana Stojanovska, Gerasimos Aravantinos, Ippokratis Korantzis, Alexandros Ioannou, Ioannis Varthalitis, Dimitrios Doufexis, Michail Nikolaou, Georgios Lypas, Iliada Bompolaki, Athina Christopoulou, Michael Liontos, Nikolaos Tsoukalas, Davide Mauri, Nikolaos Xenidis, Panagiotis Katsaounis, Georgios Oikonomopoulos, Ioannis Boukovinas

**Affiliations:** Hellenic Society of Medical Oncology (HeSMO), Athens, Greece

## Abstract

**Background:**

During the last decade, significant improvement was made in systemic therapy of pancreatic adenocarcinoma (PAC). The impact of this progress in everyday clinical practice has not been fully described yet. The aim of the study was to investigate the pattern followed by Greek Medical Oncologists regarding the treatment of patients with PAC.

**Methods:**

This observational, noninterventional multicenter study recorded clinical data from the files of 200 active patients (alive and under treatment or follow-up) for a two-year period (November 2015 until November 2017) from 20 oncology centers around Greece.

**Results:**

In total, 51 (25.5%) patients underwent radical surgical resection of PAC, and 40 (78.4%) of them received adjuvant and 1 (2.0%) neoadjuvant chemotherapy. The median time to recurrence was 7.9 months, and median overall survival (OS), 20.2 months. First-line chemotherapy was administered to 193 (96.5%) patients. The majority of patients were treated with the combination of nab-paclitaxel-gemcitabine (NPG), 5-fluorouracil, leucovorin, irinotecan, oxaliplatin (FOLFIRINOX), or gemcitabine monotherapy. Of them, 39.5% responded to the treatment. Median OS and PFS were 14.1 months and 7.0 months, respectively. Second-line treatment was administered to 112 patients. The majority received NPG, FOLFIRINOX/capecitabine, oxaliplatin, irinotecan (CAPOXIRI), or 5-fluorouracil, leucovorin, oxaliplatin (FOLFOX)/capecitabine, oxaliplatin (CAPOX). Median OS with second-line treatment was 8.6 months, and median PFS, 5.5 months. The most common chemotherapy sequences were NPG as first-line followed by FOLFIRINOX/CAPOXIRI as second-line, NPG followed by FOLFOX/CAPOX, NPG followed by other regimens, and FOLFIRINOX/CAPOXIRI followed by NPG.

**Conclusion:**

This study described the significant improvement in prognosis of PAC patients receiving palliative chemotherapy and the relatively high rate of receipt of second-line chemotherapy, according to real-world data. However, due to the nonrandomized nature of the study, any comparison between different chemotherapy regimens should be regarded with caution.

## 1. Introduction

Pancreatic cancer is a common neoplasm in the Western world, with ductal adenocarcinoma representing the dominant histology [1]. Although pancreatic cancer represents the tenth neoplasm in men and the ninth in women in incidence, it is ranked as the fourth most lethal in both sexes [2]. Pancreatic adenocarcinoma (PAC) is characterized by especially poor prognosis due to its frequently delayed diagnosis as well as its relative chemoresistance [1]. Only 15–20% of PAC cases are surgically resectable. However, even these patients experience high relapse rate and poor prognosis [1]. Adjuvant 5-fluorouracil (5FU) or gemcitabine has improved the 5-year survival rate from 8–11% to 21–22% [3, 4], while the combination of both drugs in the form of gemcitabine-capecitabine (GemCap) further improved 5-year survival to 29% [5], and finally, adjuvant modified FOLFIRINOX offered an unprecedented 5-year survival rate of 63% [6]. Systemic therapy of locally advanced or metastatic PAC underwent a slow progress during the last 2 decades with only few randomized trials, showing a small benefit in median survival from 4.4 months with 5FU to 5.6 months with gemcitabine [7], 8.5 months with nab-paclitaxel-gemcitabine (NPG) [8], and 11.1 months with irinotecan-oxaliplatin-5FU-leucovorin (FOLFIRINOX) [9].

These large differences in survival between studies mostly reflect the heterogeneity in the quality of surgical management, medical practice, or supportive care. Also, clinical trial patients are not usually representative of the common PAC patients usually characterized by poor performance status (PS) and comorbidities. Therefore, large-scale retrospective studies are important to describe how current progress in surgical or medical treatment has influenced patient outcomes in everyday practice. This knowledge is important in order to better organize health care provision, to better inform patients regarding prognosis, and to investigate ways to improve treatment outcomes.

The aim of the study was to investigate the pattern followed by Greek medical oncologists regarding the treatment of patients with PAC. Secondarily, the study aimed to collect data about the effectiveness of the systemic therapies in terms of survival parameters. Hence, by describing the status of care of PAC in Greece, we aimed to improve the therapeutic standards.

## 2. Methods

### 2.1. Study Design

This was an observational, noninterventional multicenter study of clinical practice in PAC patients in Greece. Τhe study recorded clinical data from the files of 200 consecutive active patients (alive and under treatment or follow-up) for a two-year period (one year recruitment and one year observation), prospectively (from November 2015 until November 2017 approximately) from 20 oncology centers around Greece (4 belonging in oncology hospitals). The primary endpoint was to monitor and document the clinical practice and treatments in patients with PAC in Greece. Secondary endpoints were to assess progression-free (PFS) and overall survival (OS) in different lines of treatment.

The main inclusion criteria were patients' age 18 or older, histologically or cytologically confirmed PAC, chemotherapy for PAC, alive status, and under treatment or follow-up, while exclusion criteria were the presence of other primary tumors, metastasis to the pancreas by adenocarcinoma of other primary site, and neuroendocrine pancreatic carcinoma. All patients were staged according to the 7th edition of the American Joint Committee on Cancer staging system (AJCC7). Radiological response was assessed by RECIST 1.1 criteria. However, no central radiology review was performed.

### 2.2. Ethics

The study was approved by local ethics committees, and all patients signed informed consent.

### 2.3. Statistical Analysis Plan

Descriptive statistical analysis was performed for all study data along with epidemiology methods. Continuous and categorical variables were presented with standard descriptive statistical measures, and the results were presented in tables accordingly. Median survival was calculated by the Kaplan–Meier method and compared by the log-rank test. PFS and OS were analyzed for first- and second-line treatments, as well as for different sequences of chemotherapy regimens in first- and second-line settings. PFS was defined as the time from the date of start of first- or second-line treatment to the date of progression during first- or second-line treatment, respectively, or death from other causes. OS was defined as the time from the date of start of first- or second-line treatment to the date of death from any cause. Overall PFS was defined as the time from the date of start of first-line treatment to the date of progression during second-line treatment or death from other causes. Progression-free or alive patients were censored at the last date that they were progression free or alive. Time to recurrence was defined as the time from date of surgery to the date of recurrence or last date the patient was known to remain disease free. All tests were two sided, and statistical significance was set at *p* < 0.05. SPSS 22.0 statistical package was used for all analyses. Due to the observational character of this study, no standard statistical method was used to determine the study sample size.

## 3. Results

### 3.1. Basic Patient Characteristics

The basic patient and tumor characteristics are demonstrated in Table 1. Median age at diagnosis was 65 years (range, 18–84), and the male-to-female ratio was 1.2. The majority had a PS Eastern Cooperative Oncology Group (ECOG) score of 0 and metastatic disease (stage IV). Also, the most common organ of distant metastasis was the liver in 112 (56.0%) patients, followed by lymph nodes in 31 (15.5%) and the peritoneum in 19 (9.5%). Finally, the vast majority of patients were diagnosed with ductal adenocarcinoma, while the rest were diagnosed with rare histological subtypes, such as acinar cell, adenosquamous, squamous, and noncystic mucinous carcinoma. Eighty (40%) patients were diagnosed with PAC before the study enrolment period (November 2015).

### 3.2. Radical Surgery and Adjuvant/Neoadjuvant Treatment

Fifty-one (25.5%) patients had resectable/borderline resectable tumors and underwent radical surgical resection of PAC. The stage of these tumors is described in Table 1. Of them, 41 (80.4%) patients were treated with adjuvant and/or neoadjuvant treatment, 7 (13.7%) were diagnosed with advanced disease on postoperative imaging and underwent first-line chemotherapy, and 3 (5.9%) were followed up after operation and received first-line chemotherapy after relapsing at 11–35 months postoperatively.

Of 41 patients who received adjuvant and/or neoadjuvant treatment, 40 (78.4%) patients received adjuvant, 1 (2.0%) received neoadjuvant chemotherapy, and 4 (7.8%) patients received adjuvant radiotherapy. Among them, 37 (90.2%) relapsed. Median time to recurrence was 7.9 months (95% CI, 4.4–11.4), and median OS, 20.2 months (95% CI, 5.4–25.1). Of them, 35 patients underwent first-line chemotherapy.

### 3.3. First-Line Treatment

First-line chemotherapy was administered to 193 (96.5%) patients, including 45 out of 51 (88.2%) patients who had undergone radical surgery. Chemotherapy regimens are described in Table 2. A majority of patients were treated with the combination of NPG or FOLFIRINOX or gemcitabine monotherapy. Gemcitabine was administered as monotherapy more often in older patients with poorer PS. FOLFIRINOX/capecitabine-oxaliplatin-irinotecan (CAPOXIRI) and other more unusual regimens were administered more often in patients who relapsed after radical surgery because most of them had already received gemcitabine in the adjuvant setting (Table 3).

Of 157 (80.9%) patients who were assessed for disease response, 39.5% responded to the treatment (6 patients had complete response, and 56 patients, partial response [PR]). Stable disease (SD) was achieved by 54 (34.4%) patients, and progressive disease (PD) was observed in 41 (26.1%). The NPG regimen gave the numerically highest response rate, compared with FOLFIRINOX/CAPOXIRI, gemcitabine monotherapy, or other regimens (Table 4). Overall, the difference in response rate between regimens was statistically significant (*p*=0.025).

**Figure 1:**
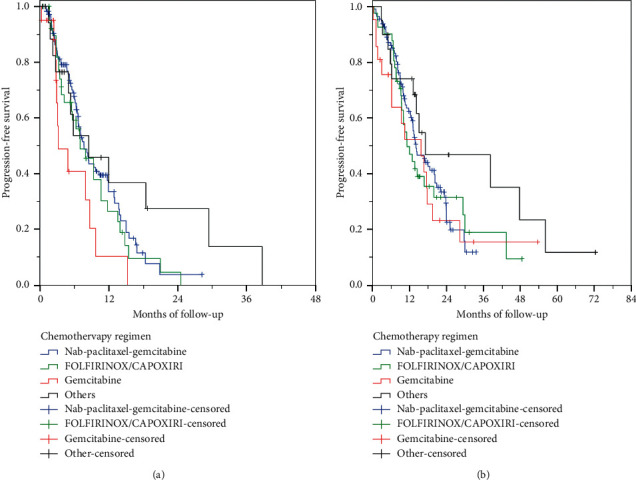
Progression-free (a) and overall survival (b) of patients receiving first-line chemotherapy according to the chemotherapy regimen. CAPOXIRI, oxaliplatin/irinotecan/capecitabine; FOLFIRINOX, oxaliplatin/irinotecan/leucovorin/5-fluorouracil.

After a median follow-up of 23.3 months (range, 0.23–72.4), 125 (64.8%) patients died and 123 (63.7%) progressed. Median OS was 14.1 months (95% CI, 11.7–16.5), and median PFS was 7.0 months (95% CI, 5.8–8.2). Median OS did not differ significantly between chemotherapy regimens (Table 5, Figure 1). In contrast, median PFS was significantly different between different chemotherapy regimens (Table 5, Figure 1). Median PFS was longer with NPG vs. gemcitabine monotherapy (*p*=0.001), FOLFIRINOX/CAPOXIRI vs. gemcitabine monotherapy (*p*=0.003), and other regimens vs. gemcitabine monotherapy (*p*=0.039) (Table 5, Figure 1). Median PFS did not differ significantly between regimens other than gemcitabine monotherapy.

Of 193 patients who received first-line chemotherapy, 77 (39.9%) were diagnosed before the study enrolment period. There was a substantial difference in survival between patients diagnosed before and those during the study enrolment period. OS of patients diagnosed before the study enrolment period was significantly longer than those diagnosed during enrolment (23.0 vs. 10.4 months, respectively, *p* < 0.001). Similarly, PFS was significantly longer in patients diagnosed before the study enrolment period (9.3 vs. 6.3 months, respectively, *p*=0.014). Median OS did not differ significantly between different chemotherapy regimens, while PFS was significantly longer with NPG and FOLFIRINOX/CAPOXIRI, in the group of patients diagnosed during study enrolment (Table 5).

### 3.4. Second-Line Treatment

In total, 112 (58.0%) patients received second-line treatment. The second-line chemotherapy regimens are shown in Table 6. The majority of patients received the combination of NPG, FOLFIRINOX/CAPOXIRI, and FOLFOX/CAPOX. Response to the treatment was assessed in 86 (76.6%) patients. A partial response was recorded in 22 (26.7%), stable disease in 32 (37.6%), and progressive disease in 31 (36.5%). Response according to the chemotherapy regimen is described in Table 7. The overall *p* value was 0.069 (trend for significance). FOLFIRINOX/CAPOXIRI demonstrated a numerically higher response rate than other regimens.

**Figure 2:**
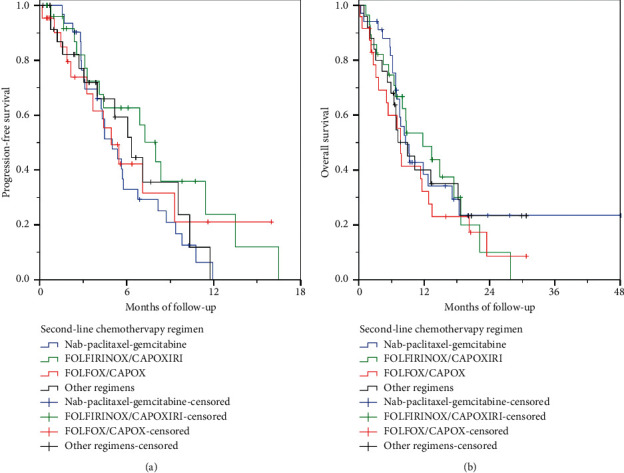
Progression-free (a) and overall survival (b) of patients receiving second-line chemotherapy according to the chemotherapy regimen. CAPOX, oxaliplatin/capecitabine; CAPOXIRI, oxaliplatin/irinotecan/capecitabine; FOLFOX, oxaliplatin/leucovorin/5-fluorouracil; FOLFIRINOX, oxaliplatin/irinotecan/leucovorin/5-fluorouracil.

After a median follow-up of 20.1 months (range, 0.2–48.1), 77 (69.4%) patients died and 67 (60.4%) progressed. Median OS was 8.6 months (95% CI 6.5–10.7), and median PFS was 5.5 months (95% CI 4.0–6.9 months). Patients treated with FOLFIRINOX/CAPOXIRI had numerically longer median OS than other regimens, but the difference was not significant (Table 8, Figure 2). Also, FOLFIRINOX/CAPOXIRI was associated with significantly longer median PFS than NPG (*p*=0.027) (Table 6, Figure 2).

Of 116 patients who were diagnosed during the study accrual period and were treated with first-line chemotherapy, 54 (46.6%) received second-line treatment. OS of patients diagnosed before the study enrolment period was significantly longer than those diagnosed during enrolment (11.3 vs. 7.6 months, respectively, *p*=0.045). In contrast, median PFS did not differ in patients diagnosed before vs. those during the study enrolment period (6.8 vs. 5.0 months, respectively, *p*=0.810). Median OS and PFS did not differ significantly between different chemotherapy regimens (Table 8).

### 3.5. Comparison of Different Chemotherapy Sequences

**Figure 3:**
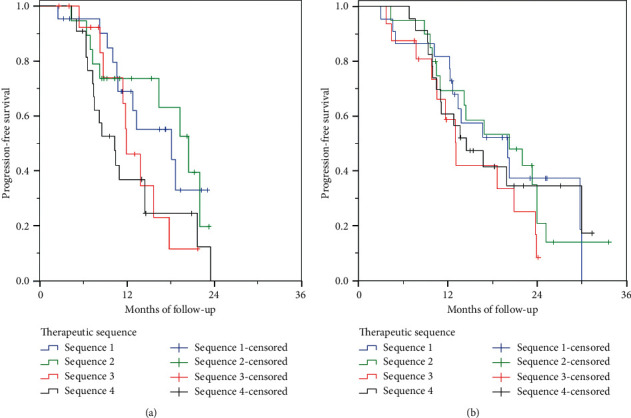
Progression-free (a) and overall survival (b) of patients according to the sequence chemotherapy regimens as first- and second-line treatments. Sequence 1 (*N* = 22): nab-paclitaxel/gemcitabine as first-line, followed by FOLFIRINOX/XELOXIRI as second-line treatment. Sequence 2 (*N* = 19): nab-paclitaxel/gemcitabine as first-line, followed by FOLFOX/CAPOX as second-line treatment. Sequence 3 (*N* = 14): nab-paclitaxel/gemcitabine as first-line, followed by other regimens as second-line treatment. Sequence 4 (*N* = 14): FOLFIRINOX/CAPOXIRI as first-line, followed by nab-paclitaxel/gemcitabine as second-line treatment.

We compared the most common sequences of chemotherapy regimens (Figure 3). These were NPG as first-line followed by FOLFIRINOX/CAPOXIRI as second-line (Sequence 1, *N* = 22), the NPG as first-line followed by FOLFOX/CAPOX as second-line (Sequence 2, *N* = 20), the NPG as first-line followed by other regimens as second-line (Sequence 3, *N* = 16), and the FOLFIRINOX/CAPOXIRI as first-line followed by NPG as second-line (Sequence 4, *N* = 24). All the other sequences comprised a maximum of 7 patients each and thus were excluded from the analysis due to their small sample size. No significant difference in median OS and overall PFS was observed between the chemotherapy sequences (Figure 3, Table 9). Sequence 1 and Sequence 2 compared with Sequence 4 demonstrated a trend for statistically longer median overall PFS (*p*=0.044 and *p*=0.054, respectively). No separate analysis of patients diagnosed during the enrolment period was performed because of their small sample size.

## 4. Discussion

The aim of the present prospective observational study was to describe the current medical practice in the treatment of PAC in Greece. To the best of our knowledge, this is one of the largest studies and the first prospective observational study in Greece. The reason for conducting this study was the difference that is often observed between clinical trial results and real-world practice data.

Results from large-scale registries of patients with PAC showed a median survival of only 2.5 months for stage III-IV disease [10–12]. These devastating figures could be explained by the fact that a low proportion of patients received palliative chemotherapy [10, 11, 13–15]. Contrarily, large registries including only patients treated with palliative chemotherapy demonstrated better survival rates. Registries in years 2007–2011 reported a median survival of 5-6 months [12, 16], while those published few years later demonstrated even better median survival times of approximately 7–10 months [13, 14].

The present study prospectively enrolled patients who were fit enough to receive palliative chemotherapy and showed a relatively long median survival of approximately 14 months However, after excluding patients diagnosed with PAC before the study accrual period, median survival was reduced to approximately 10 months, which is comparable to the survival rates reported in large prospective randomized trials, namely MPACT [8] and PRODIGE4/ACCORD11 [9]. Also, two meta-analyses of studies with FOLFIRINOX reported similar median survival of 11 months [17, 18]. To our knowledge, the largest prospective clinical cohort study published so far [19], including 1174 patients with advanced unresectable PAC, showed median PFS of 4.6 months with first-line gemcitabine monotherapy, 5.6 months with NPG, and 6.3 months with FOLFIRINOX, and a median OS of 6.8 months with first-line gemcitabine monotherapy, 9.1 months with NPG, and 11.3 months with FOLFIRINOX, which are very similar to the respective large randomized trials [7–9]. Also, a Greek group reported a median survival of 10 months with a modified biweekly NPG regimen in patients treated for advanced PAC in years 2014–2017 [20].

In the current study, more than half of patients received first-line NPG and achieved an impressive median survival of 14.1 months much longer than MPACT [8]. Even after excluding patients diagnosed before study enrolment, median survival remained at high levels (12.3 months). In contrast, median survival with FOLFIRINOX/CAPOXIRI was similar to PRODIGE4/ACCORD11 results [9], but shorter than NPG. Median survival remained similar (11.1 months, 95% CI, 7.0–15.2, data not shown in Results), when the 6 patients treated with CAPOXIRI where excluded. Notably, FOLFIRINOX is not licensed for treating PAC in Greece and needs special approval by regulatory authorities, thus limiting its availability as a treatment option. This limitation explains the reason that only a small proportion of patients received first-line FOLFIRINOX/CAPOXIRI. Finally, in the present study, the groups that received gemcitabine monotherapy or other regimens were too small to make firm conclusions.

Imbalances in basic characteristics between treatment arms might explain differences in survival. Of note, although patients treated with first-line NPG were older and had more frequently newly diagnosed metastatic disease than patients on FOLFIRINOX/CAPOXIRI, they had numerically better survival. Moreover, despite patients treated with first-line gemcitabine monotherapy were more frequently older and of poorer PS, their median survival was better than expected. Interestingly, their median PFS was disproportionately short compared with their OS, most probably, because they benefited from second-line treatment. Specifically, of 16 patients who had received first-line gemcitabine, 7 proceeded to second-line treatment: 2 with NPG, 1 with FOLFOX, 2 with cisplatin or oxaliplatin and gemcitabine, and 2 with continuation of gemcitabine after progression. Most of them had a baseline PS ECOG score of 1 before starting first-line gemcitabine.

The present study also provided information on second-line chemotherapy results. Only few prospective studies have been published. The best studied is second-line chemotherapy after gemcitabine-based chemotherapy failure. The main options are fluoropyrimidine monotherapy, fluoropyrimidine combined with oxaliplatin, irinotecan, or both. Combination chemotherapy has generally demonstrated better results than single fluoropyrimidine. A meta-analysis of clinical trials showed that there are stronger data supporting second-line irinotecan-fluoropyrimidine than oxaliplatin-fluoropyrimidine combinations [21]. An AGEO prospective study of second-line chemotherapy after first-line NPG showed that FOLFIRI (compared with FOLFOX and FOLFIRINOX) had numerically the best results in PFS (6.6 vs. 2 vs. 3.4 months, respectively) and OS (9.7 vs. 3.5 vs. 6.1 months, respectively), although differences were not significant [22]. Of note, a large prospective randomized trial NAPOLI-1 demonstrated a prolongation in median survival from 4.2 months with 5FU to 6.1 months with liposomal irinotecan as second-line treatment in PAC [23]. Two phase II studies of second-line chemotherapy with FOLFIRINOX after progression with gemcitabine-based chemotherapy led to median PFS of 3.8–5.8 months and median OS of 8.5–9.0 months [24, 25]. In the present study, the results with second-line FOLFIRINOX/CAPOXIRI compared favorably with the literature with median PFS and OS of 8 and 13 months, respectively. In conclusion, although liposomal irinotecan or irinotecan combined with fluoropyrimidine are best supported by the literature as second-line options, FOLFIRINOX has demonstrated activity that should be confirmed in the setting of prospective randomized trials.

In contrast, data on second-line gemcitabine-based chemotherapy after FOLFIRINOX failure are fewer. Gemcitabine monotherapy is an option with poor efficacy [26]. An AGEO prospective study showed better results with second-line NPG, reporting median PFS and OS of 5.1 and 8.8 months, respectively [27]. Second-line NPG after FOLFIRINOX failure was examined in a phase II trial [28]. Median PFS and OS were 3.8 and 7.6 months. Second-line NPG in the present study showed similar results with median PFS and OS of 4.5 and 8 months, respectively. Therefore, NPG is an active second-line chemotherapy regimen.

Finally, we presented the impact of different sequences of chemotherapy regimens on patient outcome. To the best of our knowledge, only few prospective studies recorded OS with specific chemotherapy sequences. AGEO prospectively recorded median survival with different chemotherapy sequences. Median OS was 10.4 months with first-line NPG followed by FOLFOX, 18.4 months with first-line NPG followed by FOLFIRI, 12.3 months with first-line NPG followed by FOLFIRINOX [22], and 18 months with first-line FOLFIRINOX followed by NPG [27]. In contrast, we showed that patients receiving NPG as first-line and FOLFIRINOX/CAPOXIRI or FOLFOX/CAPOX as second-line treatment had much longer median OS than the opposite sequence. This finding might represent useful information regarding the best therapeutic strategy or might only reflect the fact that FOLFIRINOX/CAPOXIRI is too toxic and only patients with the best prognosis can tolerate them in the second-line setting. Therefore, the validity of this finding can be confirmed only by prospective randomized trials, thus avoiding potential selection bias.

The present study has some limitations. The most important is that 40% of patients were diagnosed with PAC before the study accrual period. Thus, a selection bias of pancreatic cancer survivors might explain favorable survival results. However, the exclusion of these patients did not significantly alter the difference in survival between chemotherapy regimens. Another weakness is that only a limited number of patients received first-line FOLFIRINOX. Therefore, firm conclusions cannot be made regarding the efficacy and survival results with this regimen. Finally, the lack of quality of life and treatment toxicity data is an important limitation of this prospective study.

In conclusion, this study presented useful information regarding real-world data on systemic treatment of resected and unresectable PAC in the first- and second-line settings. An important finding is the significant improvement in prognosis of patients receiving palliative chemotherapy. However, due to the nonrandomized nature of the study any comparison between different chemotherapy regimens should be regarded with caution.

## Figures and Tables

**Table 1:** Basic patient and disease characteristics.

Characteristics		*N*	%
Age in years	<60	62	31.0
	≥60	138	69.0
Gender	Male	107	53.5
	Female	93	46.5
ECOG performance status	0	88	44.0
	1	59	29.5
	2	9	4.5
	3	3	1.5
	Not recorded	41	20.5
Histology	Ductal adenocarcinoma	186	93.0
	Acinar cell carcinoma	3	1.5
	Adenosquamous carcinoma	3	1.5
	Others	4	2.0
	Not recorded	4	2.0
Tumor stage (AJCC7)	IA	2	1.0
	IB	3	1.5
	IIA	11	5.5
	IIB	35	17.5
	III	29	14.5
	IV	118	59.0
	Not reported	2	1.0
**Total**		**200**	**100.0**

AJCC7, American Joint Committee on Cancer staging 7th edition; ECOG, Eastern Cooperative Oncology Group.

**Table 2:** Chemotherapy regimens that were administered as first-line treatment.

First-line chemotherapy regimen	*N*	%
Nab-paclitaxel/gemcitabine	113	58.5
Gemcitabine	16	8.3
Gemcitabine/5-fluorouracil	1	0.5
Gemcitabine/oxaliplatin	5	2.6
Gemcitabine/cisplatin	3	1.6
Gemcitabine/carboplatin	1	0.5
Gemcitabine/erlotinib	1	0.5
Gemcitabine/temsirolimus	1	0.5
FOLFIRINOX	36	18.7
CAPOXIRI	6	3.1
FOLFOX	4	2.1
CAPOX	2	1.0
FOLFIRI	1	0.5
CDDP/LV/5-fluorouracil	2	1.0
Cisplatin/etoposide	1	0.5
**Total**	**193**	**100.0**

CAPOX, oxaliplatin/capecitabine; CAPOXIRI, oxaliplatin/irinotecan/capecitabine; CDDP, cisplatin; FOLFIRI, irinotecan/leucovorin/5-fluorouracil; FOLFOX, oxaliplatin/leucovorin/5-fluorouracil; FOLFIRINOX, oxaliplatin/irinotecan/leucovorin/5-fluorouracil.

**Table 3:** Association of basic patient and tumor characteristics with first-line chemotherapy regimen.

Characteristics			Chemotherapy regimen				
			Nab-paclitaxel-gemcitabine	FOLFIRINOX/CAPOXIRI	Gemcitabine	Others	*p* value
Age (years)	<70	*N* (%)	70	36	7	18	0.003
			61.9	85.7	43.8	81.8	
	≥70	*N* (%)	43	6	9	4	
			38.1	14.3	56.2	18.2	
Sex	Male	*N* (%)	59	26	6	14	0.287
			52.2	61.9	37.5	63.6	
	Female	*N* (%)	54	16	10	8	
			47.8	38.1	62.5	36.4	
ECOG PS	0	*N* (%)	49	22	1	12	<0.001
			55.0	62.9	8.3	70.6	
	1	*N* (%)	37	12	5	4	
			41.6	34.2	41.7	23.5	
	2-3	*N* (%)	3	1	6	1	
			3.4	2.9	50.0	5.9	
Tumor stage	III	*N* (%)	15	4	4	1	0.010
			13.5	10.0	25.0	4.5	
	IV	*N* (%)	74	19	12	13	
			66.7	47.5	75.0	59.1	
	Relapse	*N* (%)	22	17	0	8	
			19.8	42.5	0.0	36.4	
**Total**	***N* (%)**	**111**	**40**	**16**	**22**		
		**100.0%**	**100.0**	**100.0**	**100.0**		

**Table 4:** Response to first-line treatment according to the chemotherapy regimen.

Response		First-line chemotherapy regimen				Total
		Nab-paclitaxel-gemcitabine	FOLFIRINOX/CAPOXIRI	Gemcitabine	Others	
CR	*N* (%)	5	1	0	0	6
		5.5	2.6	0.0	0.0	3.8
PR	*N* (%)	39	12	1	4	56
		42.9	31.6	10.0	22.2	35.7
SD	*N* (%)	28	13	3	10	54
		30.8	34.2	30.0	55.6	34.4
PD	*N* (%)	19	12	6	4	41
		20.9	31.6	60.0	22.2	26.1
**Total**	***N* (%)**	**91**	**38**	**10**	**18**	**157**
		**58.0**	**24.2**	**6.4**	**11.4**	**100.0**

CAPOXIRI, oxaliplatin/irinotecan/capecitabine; CR, complete response; FOLFIRINOX, oxaliplatin/irinotecan/leucovorin/5-fluorouracil; PD, progressive disease; PR, partial response; SD, stable disease.

**Table 5:** Overall and progression-free survival according to first-line chemotherapy regimen in the overall population and the group of patients diagnosed during study enrolment.

	Overall population			Diagnosed during enrolment		
	Median	95% CI	*p* value	Median	95% CI	*p* value
OS (months)						
Nab-paclitaxel-gemcitabine	14.1	10.6–17.6	0.429	12.3	8.8–15.9	0.125
FOLFIRINOX/CAPOXIRI	11.1	7.7–14.6		9.9	6.4–13.5	
Gemcitabine	10.4	3.0–17.7		6.1	0–14.8	
Other regimens	17.1	6.6–27.6		13.2	3.4–23.0	

PFS (months)						
Nab-paclitaxel-gemcitabine	7.6	6.2–9.0	0.004	6.7	6.0–7.3	0.001
FOLFIRINOX/CAPOXIRI	6.9	4.1–9.7		6.3	4.6–8.0	
Gemcitabine	2.9	2.7–3.0		2.9	2.5–3.2	
Other regimens	7.0	3.2–10.8		5.3	1.2–9.4	

CAPOXIRI, oxaliplatin/irinotecan/capecitabine; 95% CI, 95% confidence intervals; FOLFIRINOX, oxaliplatin/irinotecan/leucovorin/5-fluorouracil; OS, overall survival; PFS, progression-free survival.

**Table 6:** Chemotherapy regimens that were administered as second-line treatment.

Second-line chemotherapy regimen	*N*	%
Nab-paclitaxel/gemcitabine	36	32.1
FOLFIRINOX	27	24.1
CAPOXIRI	1	0.9
FOLFOX	18	16.1
CAPOX	4	3.6
Gemcitabine	6	5.4
Capecitabine	2	1.8
FOLFIRI	6	5.4
Gemcitabine/oxaliplatin	6	5.4
Gemcitabine/capecitabine	2	1.8
Cisplatin/gemcitabine	1	0.9
Cisplatin/irinotecan	1	0.9
Nab-paclitaxel	2	1.8
Total	112	100.0

CAPOX, oxaliplatin/capecitabine; CAPOXIRI, oxaliplatin/irinotecan/capecitabine; FOLFIRI, irinotecan/leucovorin/5-fluorouracil; FOLFOX, oxaliplatin/leucovorin/5-fluorouracil; FOLFIRINOX, oxaliplatin/irinotecan/leucovorin/5-fluorouracil.

**Table 7:** Response to second-line treatment according to the chemotherapy regimen.

Response		Second-line chemotherapy regimen				Total
		Nab-paclitaxel/Gemcitabine	FOLFIRINOX/CAPOXIRI	FOLFOX/CAPOX	Other regimens	
PR	*N* (%)	8	9	1	3	22
		25.0	45.0	6.3	23.5	25.9
SD	*N* (%)	12	6	7	7	32
		37.5	30.0	43.8	41.2	37.6
PD	*N* (%)	12	5	8	6	31
		37.5	25.0	50.0	35.3	36.5
Total	*N* (%)	32	20	16	17	85
		37.6	23.5	18.8	20.0	100.0

CAPOX, oxaliplatin/capecitabine; CAPOXIRI, oxaliplatin/irinotecan/capecitabine; FOLFOX, oxaliplatin/leucovorin/5-fluorouracil; FOLFIRINOX, oxaliplatin/irinotecan/leucovorin/5-fluorouracil; PD, progressive disease; PR, partial response; SD, stable disease.

**Table 8:** Overall and progression-free survival according to second-line chemotherapy regimen in the overall population and the group of patients diagnosed during study enrolment.

	Overall population			Diagnosed during enrolment		
	Median	95% CI	*p* value	Median	95% CI	*p* value
OS (months)						
Nab-paclitaxel-gemcitabine	8.3	6.0–10.7	0.785	7.5	6.1–8.9	0.751
FOLFIRINOX/CAPOXIRI	13.4	5.8–21.0		11.8	4.3–19.3	
FOLFOX/CAPOX	7.6	6.5–8.8		7.6	1.8–13.4	
Other regimens	8.9	5.2–12.6		6.5	2.7–10.2	

PFS (months)						
Nab-paclitaxel-gemcitabine	4.5	3.7–5.3	0.194	4.5	3.6–5.4	0.584
FOLFIRINOX/CAPOXIRI	8.0	6.4–9.6		6.9	0.5–13.3	
Gemcitabine	4.9	2.8–7.0		4.4	1.6–7.2	
Other regimens	6.3	4.4–8.3		6.1	2.2–9.9	

CAPOXIRI, oxaliplatin/irinotecan/capecitabine; 95% CI, 95% confidence intervals; FOLFIRINOX, oxaliplatin/irinotecan/leucovorin/5-fluorouracil; OS, overall survival; PFS; progression-free survival.

**Table 9:** Overall survival and overall progression-free survival according to second-line chemotherapy regimen in the overall population and the group of patients diagnosed during study enrolment.

	Median	95% CI	*p* value
OS (months)			
Sequence 1	20.0	8.7–31.4	0.523
Sequence 2	20.2	10.0–30.5	
Sequence 3	13.1	10.8–15.3	
Sequence 4	13.6	7.6–19.7	

PFS (months)			
Sequence 1	18.1	6.3–29.9	0.128
Sequence 2	20.4	15.2–25.7	
Sequence 3	11.9	9.0–14.8	
Sequence 4	10.3	7.1–13.5	

Sequence 1 (*N* = 22): nab-paclitaxel/gemcitabine as first-line followed by FOLFIRINOX/XELOXIRI as second-line treatment. Sequence 2 (*N* = 19): nab-paclitaxel/gemcitabine as first-line followed by FOLFOX/CAPOX as second-line treatment. Sequence 3 (*N* = 14): nab-paclitaxel/gemcitabine as first-line followed by other regimens as second-line treatment. Sequence 4 (*N* = 14): FOLFIRINOX/CAPOXIRI as first-line followed by nab-paclitaxel/gemcitabine as second-line treatment; 95% CI, 95% confidence intervals; OS, overall survival; PFS, progression-free survival.

## Data Availability

The data used to support the findings of this study are included within the article, and the data sources are available from the corresponding author upon request.

## References

[1] Kamisawa T., Wood L. D., Itoi T., Takaori K. (2016). Pancreatic cancer. *The Lancet*.

[2] Siegel R. L., Miller K. D., Jemal A. (2019). Cancer statistics, 2019. *CA: A Cancer Journal for Clinicians*.

[3] Neoptolemos J. P., Stocken D. D., Friess H. (2004). A randomized trial of chemoradiotherapy and chemotherapy after resection of pancreatic cancer. *New England Journal of Medicine*.

[4] Oettle H., Post S., Neuhaus P. (2007). Adjuvant chemotherapy with gemcitabine vs observation in patients undergoing curative-intent resection of pancreatic cancer. *JAMA*.

[5] Neoptolemos J. P., Palmer D. H., Ghaneh P. (2017). Comparison of adjuvant gemcitabine and capecitabine with gemcitabine monotherapy in patients with resected pancreatic cancer (ESPAC-4): a multicentre, open-label, randomised, phase 3 trial. *The Lancet*.

[6] Conroy T., Hammel P., Hebbar M. (2018). FOLFIRINOX or gemcitabine as adjuvant therapy for pancreatic cancer. *New England Journal of Medicine*.

[7] Burris H. A., Moore M. J., Andersen J. (1997). Improvements in survival and clinical benefit with gemcitabine as first-line therapy for patients with advanced pancreas cancer: a randomized trial. *Journal of Clinical Oncology*.

[8] Von Hoff D. D., Ervin T., Arena F. P. (2013). Increased survival in pancreatic cancer with nab-paclitaxel plus gemcitabine. *New England Journal of Medicine*.

[9] Conroy T., Desseigne F., Ychou M. (2011). FOLFIRINOX versus gemcitabine for metastatic pancreatic cancer. *New England Journal of Medicine*.

[10] Bernards N., Haj Mohammad N., Creemers G.-J., de Hingh I. H. J. T., van Laarhoven H. W. M., Lemmens V. E. P. P. (2015). Ten weeks to live: a population-based study on treatment and survival of patients with metastatic pancreatic cancer in the south of The Netherlands. *Acta Oncologica*.

[11] Burmeister E. A., OʼConnell D. L., Beesley V. L. (2015). Describing patterns of care in pancreatic cancer. *Pancreas*.

[12] Haj Mohammad N., Bernards N., Besselink M. G. H. (2016). Volume matters in the systemic treatment of metastatic pancreatic cancer: a population-based study in The Netherlands. *Journal of Cancer Research and Clinical Oncology*.

[13] Zijlstra M., van der Geest L. G. M., van Laarhoven H. W. M., Lemmens V. E. P. P., van de Poll-Franse L. V., Raijmakers N. J. H. (2018). Patient characteristics and treatment considerations in pancreatic cancer: a population based study in The Netherlands. *Acta Oncologica*.

[14] Miller-Ocuin J. L., Zenati M. S., Ocuin L. M. (2017). Failure to treat: audit of an institutional cancer registry database at a large comprehensive cancer center reveals factors affecting the treatment of pancreatic cancer. *Annals of Surgical Oncology*.

[15] Huang L., Jansen L., Balavarca Y. (2018). Nonsurgical therapies for resected and unresected pancreatic cancer in Europe and USA in 2003-2014: a large international population-based study. *International Journal of Cancer*.

[16] Enewold L., Harlan L. C., Tucker T., McKenzie S. (2015). Pancreatic cancer in the USA: persistence of undertreatment and poor outcome. *Journal of Gastrointestinal Cancer*.

[17] Usón Junior P. L. S., Rother E. T., Maluf F. C., Bugano D. D. G. (2018). Meta-analysis of modified FOLFIRINOX regimens for patients with metastatic pancreatic cancer. *Clinical Colorectal Cancer*.

[18] Tong H., Fan Z., Liu B., Lu T. (2018). The benefits of modified FOLFIRINOX for advanced pancreatic cancer and its induced adverse events: a systematic review and meta-analysis. *Science Report*.

[19] Hegewisch-Becker S., Aldaoud A., Wolf T. (2019). Results from the prospective German TPK clinical cohort study: treatment algorithms and survival of 1,174 patients with locally advanced, inoperable, or metastatic pancreatic ductal adenocarcinoma. *International Journal of Cancer*.

[20] Kokkali S., Tripodaki E. S., Drizou M. (2018). Biweekly gemcitabine/nab-paclitaxel as first-line treatment for advanced pancreatic cancer. *In Vivo*.

[21] Sonbol M. B., Firwana B., Wang Z. (2017). Second-line treatment in patients with pancreatic ductal adenocarcinoma: a meta-analysis. *Cancer*.

[22] Pointet A.-L., Tougeron D., Pernot S. (2020). Three fluoropyrimidine-based regimens in routine clinical practice after nab-paclitaxel plus gemcitabine for metastatic pancreatic cancer: an AGEO multicenter study. *Clinics and Research in Hepatology and Gastroenterology*.

[23] Wang-Gillam A., Li C. P., Bodoky G. (2016 Feb6). Nanoliposomal irinotecan with fluorouracil and folinic acid in metastatic pancreatic cancer after previous gemcitabine-based therapy (NAPOLI-1): a global, randomised, open-label, phase 3 trial. *Lancet*.

[24] Kim J. H., Lee S.-C., Oh S. Y. (2018). Attenuated FOLFIRINOX in the salvage treatment of gemcitabine-refractory advanced pancreatic cancer: a phase II study. *Cancer Communications*.

[25] Chung M. J., Kang H., Kim H. G. (2018). Multicenter phase II trial of modified FOLFIRINOX in gemcitabine-refractory pancreatic cancer. *World Journal of Gastrointestinal Oncology*.

[26] Sarabi M., Mais L., Oussaid N., Desseigne F., Guibert P., De La Fouchardiere C. (2017). Use of gemcitabine as a second-line treatment following chemotherapy with FOLFIRINOX for metastatic pancreatic adenocarcinoma. *Oncology Letters*.

[27] Portal A., Pernot S., Tougeron D. (2015). Nab-paclitaxel plus gemcitabine for metastatic pancreatic adenocarcinoma after FOLFIRINOX failure: an AGEO prospective multicentre cohort. *British Journal of Cancer*.

[28] Mita N., Iwashita T., Uemura S. (2019). Second-line gemcitabine plus nab-paclitaxel for patients with unresectable Advanced pancreatic cancer after first-line FOLFIRINOX failure. *Journal of Clinical Medicine*.

